# Sleep disorders among educationally active elderly people in Bialystok, Poland: a cross-sectional study

**DOI:** 10.1186/s12877-019-1248-2

**Published:** 2019-08-19

**Authors:** Mateusz Cybulski, Lukasz Cybulski, Elzbieta Krajewska-Kulak, Magda Orzechowska, Urszula Cwalina, Krystyna Kowalczuk

**Affiliations:** 1 Department of Integrated Medical Care, Faculty of Health Sciences, Medical University of Bialystok, 7a M. Sklodowskiej-Curie str., 15-096 Bialystok, Poland; 2 National security student, Faculty of Social Sciences, University of Warmia and Mazury in Olsztyn, 14 Zolnierska str., 10-561 Olsztyn, Poland; 3 Department of Statistics and Medical Informatics, Faculty of Health Sciences, Medical University of Bialystok, 37 Szpitalna str., 15-295 Bialystok, Poland

**Keywords:** Athens insomnia scale, Elderly, Epworth sleepiness scale, Insomnia severity index, Older adults, Sleep, Sleep disorders

## Abstract

**Background:**

Sleep disorders in an ageing society constitute a significant public health problem. It is estimated that approximately 50% of people aged 55 years and older have trouble sleeping, including initiating and maintaining sleep.

The aim of this study was to determine the prevalence of sleep disorders in a group of educationally active elderly people living in Bialystok, Poland.

**Methods:**

The study included a total of 182 people – residents of Bialystok – aged 60 or older; 146 women (80.22%) and 36 men (19.78%). The study used three standardized psychometric scales: The Athens Insomnia Scale (AIS), The Epworth Sleepiness Scale (ESS) and The Insomnia Severity Index (ISI).

**Results:**

More than half of the respondents scored 6 or more points on the AIS, which is considered a value that indicates a high probability of insomnia symptom occurrence. A similar percentage of respondents obtained a point value on the ISI indicating the presence of insomnia. The vast majority of respondents scored below 11 points on the ESS, which means no symptoms of excessive sleepiness. There was a significant correlation between the results of the above scales in the examined group in total and also by sex.

**Conclusions:**

Sleep disorders, particularly insomnia, constitute a significant social and health problem in the group of educationally active elderly people living in Bialystok. In light of the obtained study results, it is recommended to conduct and improve existing health education programs aimed at the elderly regarding sleep disorders to improve the quality of their sleep, and thus quality of life, and raise the awareness of the elderly about the importance of sleep in everyday life. There is a need for further research in the field of sleep disorders in the elderly to determine the prevalence of these disorders on a national scale.

## Background

Sleep quality is one of the key determinants of good health. Its importance is particularly evident among older people, as sleep disorders and difficulty falling asleep become more common with age [1, 2].

Sleep disorders in an ageing society constitute a significant public health problem. It is estimated that approximately 50% of people aged 55 years and older have trouble sleeping, including initiating and maintaining sleep [3–7]. Moderate sleep disorders in the elderly are often associated with functional deficits in everyday life, including increased sense of tiredness and mood disorders, which in turn leads to lower quality of life, increased risk of depression and pathological phenomena, such as addiction to alcohol or medications [3, 6, 8–11].

The most common sleep disorder among the elderly is insomnia. The prevalence of insomnia symptoms in the elderly is between 20 and 40% [12–15]. Other studies show that the incidence of insomnia, referred to as a chronic sleep disorder, was reported in 50–70% of all people aged 65 years old [16, 17]. Similar data pertain to the Polish population. In a Polish research program on cardiovascular disease risk factors, NATPOL, in a group of almost 2500 respondents, subjective sleep difficulties were declared by 50.5% of the studied population [18]. Among women, the percentage was 58.9% and was higher than in the group of men (41.4%). Subjective insomnia was found in 50.9% of people between 60 and 79 years of age, especially among women (74.8%). Data from the NATPOL study are similar to previous epidemiological studies on sleep disorders in the Polish population [19].

In Poland, no studies have so far been carried out to assess the incidence of sleep disorders among socially active older people, in particular seniors who are educationally active. Published studies have shown that daily physical and social activity contribute to a lower incidence of sleep disorders in the elderly people [20–22]. Too short or too long sleep periods contribute to higher morbidity among seniors [22, 23]. A small number of activities performed during the day and frequent naps can affect the negative changes in the sleep-wake rhythm, and thus worsens the quality of sleep [22, 24]. Intensive physical and social activity has a positive effect on the quality, duration and efficiency of sleep [22, 25, 26].

In connection with the above, the aim of this study was to determine the prevalence of sleep disorders in a group of educationally active elderly people living in Bialystok and comparing the differences between the frequency of occurrence of sleep disorders among educationally active seniors against the background of elderly people in general. We have formulated the following research questions:

1. What is the actual prevalence of sleep disorders in the educationally active older adults in Bialystok?
2. What are the dimensions of inequalities in the sleep health of older adults, including sex, age and group of origin?
3. What are the correlations between sleep disorders and socio-demographic characteristics of the study group?

We assumed that despite the fact that the studied group consists of socially active people, preferring active and healthy ageing, the group will be characterized by an high presence of sleep disorders, particularly insomnia.

## Methods

### Study design and participants

This is a 4-month cross-sectional study of elderly persons aged 60 years and older living in urban in Bialystok. The study was conducted in 2 groups of educationally active elderly people: students of the University of the Healthy Senior (UHS) and the University of Psychogeriatric Prophylactics (UPP) (74 people, which included 66 women (89.19%) and 8 men (10.81%)) and students of the University of the Third Age in Bialystok (UTA) (108 people, which included 80 women (74.07%) and 28 men (25.93%)). The study included a total of 182 people – residents of Bialystok – aged 60 or older; 146 women and 36 men. Both groups were created by elderly people who are characterized by active aging - they are physically and socially active, take part in educational activities, etc. The students of the UTA take part in activities on diverse subjects in which there is no emphasis on health issues. Participants of the UHS and the UPP are a specific group of active seniors, because as part of the program they only carry out health-related classes, including those devoted to mental disorders, for example sleep disorders in the elderly. We decided to assess the occurrence of sleep disorders in both groups and compare them with each other to see if there are any significant differences between them for the originality of the study. In addition, respondents were asked about the occurrence of sleep disorders in the past. Earlier problems with sleep were reported by 11 respondents (6.04%). The respondents’ detailed socio-demographic data characteristics are shown in Table 1.

**Table 1:** Socio-demographic characteristics of the studied group

		UTA *n* = 108		UHS/UPP *n* = 74		Total *n* = 182	
		n	%	n	%	n	%
sex	women	80	74.07%	66	89.19%	146	80.22%
	men	28	25.93%	8	10.81%	36	19.78%
age	60–70 years	47	43.52%	51	68.92%	98	53.85%
	71–80 years	53	49.07%	21	28.38%	74	40.66%
	81–90 years	8	7.41%	2	2.70%	10	5.49%
marital status	married	43	39.81%	26	35.14%	69	37.91%
	widowed	51	47.22%	25	33.78%	76	41.76%
	single	5	4.63%	4	5.41%	9	4.95%
	divorced	7	6.48%	16	21.62%	23	12.64%
	separated	2	1.85%	3	4.05%	5	2.75%
education	higher	56	51.85%	34	45.95%	90	49.45%
	secondary	40	37.04%	32	43.24%	72	39.56%
	technical	8	7.41%	6	8.11%	14	7.69%
	vocational	3	2.78%	2	2.70%	5	2.75%
	primary	1	0.93%	0	0.00%	1	0.55%
occurrence of sleep disorders in the past	yes	4	3.70%	7	9.46%	11	6.04%
	no	104	96.30%	67	90.54%	171	93.96%

Abbreviations: *UHS* University of Healthy Senior, *UPP* University of Psychogeriatric Prophylactics, *UTA* University of the Third Age

### Procedure and ethical considerations

Of 400 individuals willing to participate, 182 (80.22% of men and 19.78% of women) met the inclusion criteria and were eligible for further analysis. Participants were selected from second conveniently selected institutions in the city of Bialystok, conducting educational activities for the elderly. After institutions selection, we spread information about the study using flyers to inform future participants about the main aim and hypothesis. The inclusion criteria were: (1) age ≥ 60, (2) place of residence in Bialystok, (3) be free of cognitive diseases (the information about their cognitive functioning was confirmed through evidence provided by Clock-Drawing Test), (4) be able to read and write individually, and (5) written consent to participate in the study.

Participant selection was intentional. For the study to be representative, the authors assumed collecting at least 150 fully completed questionnaires, 75 in each studied group. More copies of the research tool were distributed, however, not all of the questionnaires were returned to the authors. In the group of UHS and UPP students, 150 questionnaires were distributed (50.00% response rate), while 250 questionnaires were given out to the group of UTA students (42.80% response rate). Respondents received paper copies of the questionnaire, which they filled out at home after receiving detailed information about filling out the questionnaire from members of the study team. They returned completed questionnaires before the next lecture at their university). Each participant of the study could withdraw from it at any time.

The study was conducted from February to June 2018. The research conformed with the Good Clinical Practice guidelines and the procedures were in accordance with the Helsinki Declaration of 1975, as revised in 2000 (concerning the ethical principles for medical research involving human subjects and prohibiting the provision of patient’s name, initials or hospital evidence number) and with the ethical standards of the institutional committee on human experimentation (statute from the Bioethics Committee of the Medical University in Bialystok no. R-I-002/172/2018). The members of the research team gave written and verbal information about the study to potential participants. They received the information about the project and gave written consent to participate.

### Measures

#### Athens insomnia scale – AIS

The Athens Insomnia Scale (AIS) is a short self-report tool consisting of eight statements pertaining various insomnia symptoms, allowing for their quantitative measurement based on ICD-10 criteria [27, 28]. Each item on the AIS is rated by the respondent on a scale of 0–3 points, where 0 means lack of a given symptom and 3 indicates intensified symptom severity. The total score on the AIS is between 0 and 24 points. The first five items refer to sleep-related symptoms (difficulties falling asleep, waking up at night, waking up early in the morning, sleep duration and quality) and correspond to criterion A of nonorganic insomnia according to ICD-10. A given symptom should be marked if it occurred at least three times a week for at least a month, which is consistent with the duration and frequency of symptoms (criterion B) required for diagnosing insomnia in ICD-10. The other three items are related to daytime functioning (well-being, physical and mental acuity, drowsiness) and correspond to criterion C for insomnia diagnosis according to ICD-10, which includes complaints about the consequences of insomnia during the day [29, 30].

The original validation studies showed the high reliability and accuracy of this tool. A total AIS score of 6 and more points is considered to be a value that allows to conclude with a high probability about the occurrence of insomnia (93% sensitivity, 85% specificity) [27, 28].

AIS is one of the most frequently used scales, both for diagnostic purposes and in studies on the effectiveness of insomnia treatment [31, 32].

#### Epworth sleepiness scale – ESS

The Epworth Sleepiness Scale (ESS) is a self-assessment questionnaire consisting of eight questions, the aim of which is to assess the likelihood of falling asleep in eight everyday life situations, such as watching TV, eating meals, or driving a car on a scale of 0 to 3. The ESS score can be from 0 to 24 points. A score below 10 points indicates a lack of excessive sleepiness, while a score over 10 points indicates pathological sleepiness that should be assessed by a doctor. The questionnaire duration is about 3 min [33]. Internal consistency using Cronbach’s alpha ranges from 0.73 to 0.90 [33, 34].

#### Insomnia severity index – ISI

The Insomnia Severity Index (ISI) is used for subjective assessment of sleep disorders in the last 2 weeks. It consists of 7 questions, one each in the following categories: 1. difficulty falling asleep, 2. difficulty staying asleep, 3. problems due to waking up too early, 4. assessment of satisfaction with quality of sleep, 5. impact of sleep disorders on daily functioning, 6. noticeability of sleep problems by others, 7. degree of concern about the sleep disorder. Each question has 5 possible answers on the Likert scale (0 – no problem, 4 – very serious problem) [35]. The ISI result is the sum of the answers provided to all the questions. One can obtain from 0 to 28 points on the ISI. The results are interpreted as follows: 0–7 points – lack of clinically significant sleep disorders; 8–14 points – subthreshold insomnia; 15–21 points – clinically significant insomnia of moderate severity; 22–28 points – severe insomnia [36]. The sensitivity and specificity of the tool is 94% [36]. The scale has good reliability – internal consistency based on Cronbach’s alpha ranges from 0.74 to 0.78 [35].

### Statistical analysis

Data were prepared using Microsoft Excel 2013 and statistical analysis using STATISTICA 13.3 software. The following descriptive statistics were used to describe the quantitative variables: arithmetic mean, standard deviation, and median. The Shapiro-Wilk test was used to assess the normality of distribution of the quantitative variables. Normal distribution of the quantitative variables was not found. The Mann-Whitney test was used to compare two groups, and the Kruskal-Wallis test was used in cases of more than two groups. Spearman’s rank correlation coefficient was used to evaluate correlations between quantitative variables. The results were considered statistically significant at *p* < 0.05.

## Results

Average AIS score for study sample was 7.20. More than half of the respondents (53.85%) had a AIS score ≥ 6, suggestive of insomnia symptom occurrence. Average ESS score for participants was 6.34. The vast majority of respondents (82.97%) had a ESS score < 10, which means no symptoms of excessive sleepiness. Average ISI score for respondents was 8.86. More than half of the respondents (52.76%) had a ISI score ≥ 8, which indicate the presence of insomnia (Tables 2 and 3).

**Table 2:** Descriptive statistics for AIS, ESS, and ISI

scale	$\overline{x}$	Me	Min	Max	SD
AIS	7.20	6	0	24	4.73
ESS	6.34	5.5	0	23	4.44
ISI	8.86	8	0	26	5.64

Abbreviations: *AIS* Athens Insomnia Scale, *ESS* Epworth Sleepiness Scale, *ISI* Insomnia Severity Index, $\overline{x}$ mean, *Max* maximum, *Me* median, *Min* minimum, *SD* standard deviation

**Table 3:** The obtained point values on the AIS, ESS, and ISI taking into account threshold values allowing to recognize sleep disorders

scale	point values	n	%
AIS	0–5	84	46.15%
	6–24	98	53.85%
ESS	0–10	151	82.97%
	11–24	31	17.03%
ISI	0–7	86	47.25%
	8–14	155	37.91%
	15–21	180	13.74%
	22–28	182	1.10%

Abbreviations: *AIS* Athens Insomnia Scale, *ESS* Epworth Sleepiness Scale, *ISI* Insomnia Severity Index

There were no significant associations with origin (Table 4), sex (Table 5), age (Table 6), marital status and level of education and sleep disorders.

**Table 4:** Comparison of descriptive values of the AIS, ESS, and ISI with the respondents’ group of origin

scale	UTA *n* = 108			UHS/UPP *n* = 74			*p*
	$\overline{x}$	SD	Me	$\overline{x}$	SD	Me	
AIS	7.04	4.54	6	7.43	5.01	6	0.738
ESS	6.37	4.88	5	6.28	3.75	6	0.529
ISI	8.71	5.78	8	9.08	5.47	8	0.669

Abbreviations: *AIS* Athens Insomnia Scale, *ESS* Epworth Sleepiness Scale, *ISI* Insomnia Severity Index, $\overline{x}$ mean, *Me* median, *p p*-value, *SD* standard deviation, *UHS* University of Healthy Senior, *UPP* University of Psychogeriatric Prophylactics, *UTA* University of the Third Age

**Table 5:** Comparison of descriptive values of the AIS, ESS, and ISI with the respondents’ sex

scale	women *n* = 146			men *n* = 36			*p*
	$\overline{x}$	SD	Me	$\overline{x}$	SD	Me	
AIS	7.10	4.70	6	7.61	4.88	8	0.475
ESS	6.19	4.29	5	6.92	5.04	6	0.561
ISI	8.79	5.55	8	9.14	6.06	8	0.727

Abbreviations: *AIS* Athens Insomnia Scale, *ESS* Epworth Sleepiness Scale, *ISI* Insomnia Severity Index, $\overline{x}$ mean, *Me* median, *p p*-value, *SD* standard deviation, *UHS* University of Healthy Senior, *UPP* University of Psychogeriatric Prophylactics, *UTA* University of the Third Age

**Table 6:** Comparison of descriptive values of the AIS, ESS, and ISI with the respondents’ age

scale	60–70 years (I)			71–80 years (II)			81–90 years (III)			*p*	post-hoc		
	$\overline{x}$	SD	Me	$\overline{x}$	SD	Me	$\overline{x}$	SD	Me		I-II	I-III	II-III
AIS	6.79	4.89	5.0	7.47	4.39	7.0	9.20	5.37	8.5	0.156	–	–	–
ESS	6.06	3.86	5.5	6.70	5.18	5.5	6.30	4.06	5.5	0.956	–	–	–
ISI	8.51	5.72	7.0	8.99	5.51	9.0	11.40	5.72	13.0	0.288	–	–	–

Abbreviations: *AIS* Athens Insomnia Scale, *ESS* Epworth Sleepiness Scale, *ISI* Insomnia Severity Index, $\overline{x}$ mean, *Me* median, *p p*-value, *SD* standard deviation

There was a significant correlation between the occurrence of insomnia symptoms measured using the AIS and the ISI and the occurrence of symptoms of excessive sleepiness measured by the ESS in the examined group in total and also by sex. In the UTA group there were statistically significant correlations between all the studied scales, while in the UHS group the only correlation coefficient that was statistically significant was between AIS and ISI. In the groups aged 60–70 and 71–80 years, all coefficients were statistically significant, while in the oldest group only the relationship between AIS and ISI proved to be significant (Tables 7, 8, 9 and 10).

**Table 7:** Spearman’s rank correlation coefficient between the AIS, ESS, and ISI results in the studied group in general

	AIS		ESS		ISI	
	r_s_	*p*	r_s_	*p*	r_s_	*p*
AIS	–	–	0.261	< 0.001*	0.795	< 0.001*
ESS	0.261	< 0.001*	–	–	0.331	< 0.001*
ISI	0.795	< 0.001*	0.331	< 0.001*	–	–

Abbreviations: *AIS* Athens Insomnia Scale, *ESS* Epworth Sleepiness Scale, *ISI* Insomnia Severity Index, *r*_*s*_ Spearman’s rank correlation coefficient, *p p*-value, * statistically significant value

**Table 8:** Spearman’s rank correlation coefficient between the AIS, ESS, and ISI results and the respondents’ group of origin

	UTA						UHS/UPP					
	AIS		ESS		ISI		AIS		ESS		ISI	
	r_s_	*p*	r_s_	*p*	r_s_	*p*	r_s_	*p*	r_s_	*p*	r_s_	*p*
AIS	–	–	0.339	< 0.001*	0.794	< 0.001*	–	–	0.145	0.217	0.794	< 0.001*
ESS	0.339	< 0.001*	–	–	0.405	< 0.001*	0.145	0.217	–	–	0.212	0.070
ISI	0.794	< 0.001*	0.405	< 0.001*	–	–	0.794	< 0.001*	0.212	0.070	–	–

Abbreviations: *AIS* Athens Insomnia Scale, *ESS* Epworth Sleepiness Scale, *ISI* Insomnia Severity Index, *r*_*s*_ Spearman’s rank correlation coefficient, *p p*-value, *UHS* University of Healthy Senior, *UPP* University of Psychogeriatric Prophylactics, *UTA* University of the Third Age, * statistically significant value

**Table 9:** Spearman’s rank correlation coefficient between the AIS, ESS, and ISI results and the respondents’ sex

	men						women					
	AIS		ESS		ISI		AIS		ESS		ISI	
	r_s_	*p*	r_s_	*p*	r_s_	*p*	r_s_	*p*	r_s_	*p*	r_s_	*p*
AIS	–	–	0.241	0.003*	0.800	< 0.001*	–	–	0.341	0.042*	0.744	< 0.001*
ESS	0.241	0.003*	–	–	0.287	< 0.001*	0.341	0.042*	–	–	0.428	0.009*
ISI	0.800	< 0.001*	0.287	< 0.001*	–	–	0.744	< 0.001*	0.428	0.009*	–	–

Abbreviations: *AIS* Athens Insomnia Scale, *ESS* Epworth Sleepiness Scale, *ISI* Insomnia Severity Index, *r*_*s*_ Spearman’s rank correlation coefficient, *p p*-value, * statistically significant value

**Table 10:** Spearman’s rank correlation coefficient between the AIS, ESS, and ISI results and the respondents’ age

	60–70 years						71–80 years						81–90 years					
	AIS		ESS		ISI		AIS		ESS		ISI		AIS		ESS		ISI	
	r_s_	*p*	r_s_	*p*	r_s_	*p*	r_s_	*p*	r_s_	*p*	r_s_	*p*	r_s_	*p*	r_s_	*p*	r_s_	*p*
AIS	–	–	0.204	0.044*	0.799	< 0.001*	–	–	0.341	0.003*	0.812	< 0.001*	–	–	0.285	0.425	0.752	0.012*
ESS	0.204	0.044*	–	–	0.310	0.002*	0.341	0.003*	–	–	0.376	0.001*	0.285	0.425	–	–	0.143	0.693
ISI	0.799	< 0.001*	0.310	0.002*	–	–	0.812	< 0.001*	0.376	0.001*	–	–	0.752	0.012*	0.143	0.693	–	–

Abbreviations: *AIS* Athens Insomnia Scale, *ESS* Epworth Sleepiness Scale, *ISI* Insomnia Severity Index, *r*_*s*_ Spearman’s rank correlation coefficient, *p p*-value, * statistically significant value

## Discussion

In the conducted study, insomnia on the AIS was found in more than half of the elderly subjects. The average point value obtained on the AIS was 7.20 ± 4.73 points. Kim et al. [37], who included 881 Koreans aged 60 years or older in their study, estimated the prevalence of insomnia using AIS at 32.7%. In addition, the authors showed that insomnia was diagnosed less frequently in the group of people aged 80 years and older (22.4%) than in younger age groups (34.2%).

The average AIS score in the Ibáñez-del Valle et al. study [38], which included 62 elderly subjects, was 4.0 ± 4.0 points. According to the principles of interpretation of this scale’s results, 20% of the studied patients had problems with insomnia. In a group of 107 seniors studied by Abd Allah et al. [39] in the Egyptian city of Zagazig, 33.6% of the respondents had insomnia according to the AIS. The occurrence of insomnia in a group of 142 subjects studied by Hishikawa et al. [40] was 17.1%. The prevalence of insomnia in a sample of 1005 Greek citizens, studied by Paparrigopoulos et al. [41], was 25.3%, measured using the AIS. Similarly to our study, insomnia increased with age.

The conducted own research showed significant correlations between ESS and ISI as well as AIS and ISI in younger age groups, and did not show the above correlations in the oldest age group. This may have been the result of a small group of respondents in the 81–90 age group, which is this topic requires further research on a larger group in order to confirm the lack of this correlation in detail. Similar relationships were demonstrated between groups (UTA vs. UHS/UPP). This may have resulted from the fact that UHS/UPP students were educated in the field of health, including mental health and had classes on sleep disorders, while the UTA group did not have classes in this field.

A possible explanation for the differences in insomnia levels in our research and the cited studies by other authors may be differences in the level of medical care that the respondents were under, a low level of awareness among older people about specialist medical consultations regarding sleep disorders, and the co-occurrence of chronic somatic diseases. In addition, cultural differences, environmental factors, and lifestyle behaviors may explain the observed differences between the individual study results using the AIS. The incidence of insomnia among the subjects was higher than that observed in older people remaining in long-term care in a study conducted in Canada by Voyer et al. [42], which showed that the insomnia percentage was only 17.4%. This difference may result from other research tools used to assess the quality of sleep and the size of the study sample. It may also result from a higher level of care and the facilities available in long-term care centers in Western countries.

In our study, the average ESS score was 6.34 ± 4.44 points, and thus did not provide grounds for identifying excessive sleepiness in the studied seniors. Lack of excessive daytime sleepiness was reported by over 80.00% of the respondents.

In a study conducted by Brandão et al. [43], in a group of 131 elderly, the average ESS point value was 8.6 ± 2.8. In another study by Brandão et al. [44], 40 seniors (30.5%) reported excessive daytime sleepiness as measured by the ESS, while the average ESS score in the study group was 8.32 ± 2.2 points, which is not an indication of excessive daytime sleepiness, similar to other studies [45, 46]. In the study of Tsuno et al. [47], which included a population of 2184 elderly people in France, men more often scored > 10 points on the ESS compared with women – 12.0% men and 6.0% of women had excessive daytime sleepiness. In the Sforza et al. study [48], which included 232 seniors (average age: 75), the average ESS point value was 5.6 ± 3.5 points.

The observed variability in the reported prevalence rates of excessive daytime sleepiness may result to a large extent from an inconsistence of the measurements and tools used, differing definitions of the studied phenomenon, as well as the co-occurrence of chronic somatic diseases. Simultaneously, it is worth emphasizing that excessive daytime sleepiness has a negative impact on the quality of life of the elderly, because it predisposes them to taking naps, which after a longer period of time disturb the duration and quality of nighttime sleep [49].

The average ISI point value was 8.86 ± 5.64 points, which indicates the occurrence of subthreshold insomnia in the studied population. Insomnia of varying degrees of severity was found in 52.75% of the respondents. In the study by Sakamoto et al. [50], conducted in a group of 112 elderly inhabitants of the Domkhar valley (Japan), the average ISI value was 2.8 ± 3.6 points for men and 4.1 ± 4.4 points for women. Based on the analyzed scale, a significant ISI score (of 8 points or more) was recorded in 17 people (15.2%). The percentage of respondents who reported insomnia using the ISI in an elderly population of Alexandria was 33.1% [51].

Another ISI study conducted among a local community in China showed an average ISI value of 10.38 ± 5.23 points [52]. The average overall ISI score in the Dragioti et al. study [53] was 9.8 ± 5.5 points. Analysis of individual categories showed that 35.7% had no insomnia, 44.3% had subthreshold insomnia, 17.8% had moderate clinical insomnia, and 2.2% had severe clinical insomnia. This means that in general terms 20.0% of the study group suffered from clinically significant insomnia (ISI ≥ 15). Similar values were recorded in our study.

In the Pigeon et al. study [54], which included 15 seniors, the average ISI value was 15.5 ± 2.7 points. In the Tamura et al. study [55], which included 51 patients with insomnia aged 60 and over, the average ISI score was 16.94 ± 5.12 points. As in the case of the AIS, also in this case a possible explanation for the differences in insomnia levels in our own as well as the cited studies by other authors may be differences in the level of medical care, the co-occurrence of chronic somatic diseases, as well as cultural differences, environmental factors, and lifestyle.

The conducted study had several limitations. First of all, although the scales used are sensitive tools for detecting sleep disorders, false negatives cannot be ruled out. All scales focus on the subjective symptoms of the above disorders, and objective (clinical) criteria were not taken into account. Secondly, for the survey to be representative on a national scale, it should be extended to a larger group of older people from all over Poland. Thirdly, the studied group is characterized by an overrepresentation of women in relation to men. Future studies should include a larger (comparable) number of men.

## Conclusions

Sleep disorders, particularly insomnia, constitute a significant social and health problem in the group of educationally active elderly people living in Bialystok. In light of the obtained study results, it is recommended to conduct and improve existing health education programs aimed at the elderly regarding sleep disorders to improve the quality of their sleep, and thus quality of life, and raise the awareness of the elderly about the importance of sleep in everyday life. There is a need for further research in the field of sleep disorders in the elderly to determine the prevalence of these disorders on a national scale.

## Data Availability

Data supporting the findings are available upon request. Please contact the corresponding author Mateusz Cybulski (mateusz.cybulski@umb.edu.pl) for data access.

## Abbreviations

AIS: The Athens Insomnia Scale
ESS: The Epworth Sleepiness Scale
ICD-10: International Statistical Classification of Diseases and Related Health Problems 10th Revision
ISI: The Insomnia Severity Index
NATPOL: Hypertension in Poland
UHS: University of the Healthy Senior
UPP: University of Psychogeriatric Prophylactics
